# A survey and perspective on neuromorphic continual learning systems

**DOI:** 10.3389/fnins.2023.1149410

**Published:** 2023-05-04

**Authors:** Richa Mishra, Manan Suri

**Affiliations:** NVM and Neuromorphic Hardware Research Group, Department of Electrical Engineering, Indian Institute of Technology Delhi, New Delhi, India

**Keywords:** neuromorphic algorithm, hardware, lifelong learning, incremental learning, in-memory computing, digital architectures, continual learning

## Abstract

With the advent of low-power neuromorphic computing systems, new possibilities have emerged for deployment in various sectors, like healthcare and transport, that require intelligent autonomous applications. These applications require reliable low-power solutions for sequentially adapting to new relevant data without loss of learning. Neuromorphic systems are inherently inspired by biological neural networks that have the potential to offer an efficient solution toward the feat of continual learning. With increasing attention in this area, we present a first comprehensive review of state-of-the-art neuromorphic continual learning (NCL) paradigms. The significance of our study is multi-fold. We summarize the recent progress and propose a plausible roadmap for developing end-to-end NCL systems. We also attempt to identify the gap between research and the real-world deployment of NCL systems in multiple applications. We do so by assessing the recent contributions in neuromorphic continual learning at multiple levels—applications, algorithms, architectures, and hardware. We discuss the relevance of NCL systems and draw out application-specific requisites. We analyze the biological underpinnings that are used for acquiring high-level performance. At the hardware level, we assess the ability of the current neuromorphic platforms and emerging nano-device-based architectures to support these algorithms in the presence of several constraints. Further, we propose refinements to continual learning metrics for applying them to NCL systems. Finally, the review identifies gaps and possible solutions that are not yet focused upon for deploying application-specific NCL systems in real-life scenarios.

## 1. Introduction

Multiple intricate challenges are emerging as the world is moving toward incorporating automation in several sectors like transport and healthcare. In most of these applications, the task is to deal with incrementally available data in an uncontrolled environment. While neural networks today have enabled some automation, they are trained in a controlled environment with a pre-determined and limited sample set with interleaved classes. With on-chip learning, there is a scope to periodically train the network with changing data in deployed systems. However, when trained for a new class, these networks lose the previously trained information, a phenomenon termed catastrophic forgetting (McCloskey and Cohen, 1989). Much research, hence, has gone into designing systems for learning continually while averting catastrophic forgetting, with most of the approaches deeply inspired by biological neural networks (Parisi et al., 2019). While this is true pan-machine learning, neuromorphic approaches deserve special attention due to their inherent closeness with biological neural networks. With recent attention growing slowly in this direction, there is a need to systematically review the current state-of-the-art to identify the gaps between implementation and deployment in real-world applications. This study presents the first comprehensive review of neuromorphic algorithms, hardware architectures, and emerging nano-device-based solutions specifically targeting continual learning. The attempt is to identify the critical constituents of high-performing end-to-end solutions and propose a plausible roadmap for developing neuromorphic systems for continual learning.

The article is organized as follows. We discuss the significance of NCL systems for real-world applications in Section 2. In this study, we also draw out the requirements of these applications in the context of continual learning systems. In Section 3, we discuss fundamental aspects of biological systems paramount to continual learning. The hardware implementations of studies covered in this section are further discussed in Section 4. Multiple hardware-level considerations are also discussed, such as imbalanced workflow and the need for excessive reconfigurability at the hardware level. Both digital and emerging nano-device based architectures for NCL systems are covered. In Section 5, we propose modifications to the continual learning metric for the analysis of NCL systems. Finally, in Section 6, we propose a plausible roadmap for further development of the NCL systems based on our analysis of the current literature.

## 2. Applications of NCL systems

While NCL systems are relevant for various applications, in this section, we specifically focus on examples of (i) healthcare and (ii) mobility.

### 2.1. Healthcare

Spiking neural network approaches are being actively researched for healthcare applications as they promise low-power implementation critical for the application (Donati et al., 2018, 2019; Vasquez Tieck et al., 2019; Bezugam et al., 2022). Considering diagnostics of medical images, the significance of continual learning grows due to differences in imaging parameters and physiological changes in the data (Hofmanninger et al., 2020; Amrollahi et al., 2022). Implementation of spiking neural networks is also seen in prosthetic applications though adaptation to highly variant physiological signals such as EEG and EMG is not shown in these implementations (Mukhopadhyay et al., 2018; Ma et al., 2020). Some implementations use an online-unsupervised engine to generate labels for training a semi-supervised STDP-based neural network for signal processing of physiological data (Mukhopadhyay et al., 2021), yet the approach does not account for lost information once it retrains on new data, making adaptation slow and repetitive. Recent studies have proposed continual learning systems for wearable devices (Leite and Xiao, 2022). Leite and Xiao (2022) designed a dynamically expanding neural network for human activity recognition. As the subjects change, the network is required to adapt to the new style without forgetting that of the previous subject. Such applications could heavily benefit from neuromorphic approaches given the low-power computational requisite for wearable devices (Covi et al., 2021).

### 2.2. Smart mobility

Future smart vehicles require hefty cognition tasks, such as pathfinding, multi-vehicle tracking, and odometry system. All these tasks can benefit from neuromorphic continual learning algorithms for incrementally assessing and deciding in real-life situations (Chen et al., 2018, 2020). For example, changing the model of vehicles in multi-vehicle tracking on highways requires algorithms that can learn incrementally without catastrophically forgetting older models. Kim et al. (2022) utilized continual learning to provide the cognitive ability to the license plate detection system for accurate detection when the background of the image changes, while sequentially identifying and processing the numbers. Considering visual odometry, neuromorphic vision sensors have been designed (Zhu et al., 2019), utilizing the inherent enhanced edge detection capability of neuromorphic systems. Visual odometry has also evidently benefitted from continual learning approaches for deployment in drastically different environments (Vödisch et al., 2023).

From the above discussion, the significance of NCL systems is evident. Along with that, key requirements of a continual learning system can be drawn out. Functionally, the system should autonomously be able to adapt to new classes without forgetting older learned information, evident in the above applications. Hence, the system should be algorithmically robust to catastrophic forgetting. The system should also be reconfigurable in terms of network parameters such as synaptic weights, along with on-chip learning capabilities for adapting to new relevant data.

## 3. Plausible biological evidence relevant for NCL systems

Various studies in neuroscience have proposed fundamental aspects that enable the mammalian brain to learn continually. Most of them deal with encoding and retrieval methodologies of episodic memories. Keen interest is shown in these aspects by various implementations for adopting them in artificial neural networks (ANNs), yet very few have discussed the area comprehensively to identify missing cues in the vast domain of neuroscience. The discussion has been in the context of non-neuromorphic approaches, as given by Parisi et al. (2019) and Hadsell et al. (2020). We present a first analysis of these traits with the intent of adopting them into neuromorphic systems while identifying components that make a system resilient to catastrophic forgetting. Several schools of thought behind episodic memory encoding and retrieval are mentioned in the following sub-sections. The discussion also highlights the ability of the neuromorphic algorithms to self-adaptively identify the change in the input to avoid catastrophic forgetting.

### 3.1. Complementary learning system

McClelland et al. (1995) and McClelland (2013) focused on the role of the hippocampus and neocortex in continual learning, where the hippocampus was shown to adapt rapidly to the incoming data at a faster pace and the neocortex was shown to store important information at a slower pace. The neo-cortical neurons are able to extract structure from gathered experience that helps it to recreate the encoding for previously encountered stimuli. Such a setup is termed as Complementary Learning System (CLS), explained in detail by McClelland et al. (1995).

Muńoz-Mart́ın et al. (2019) emulated a complementary learning system in a hybrid supervised-unsupervised network where the pre-trained supervised feature identifier is analogous to the neo-cortical function of identification of structural information from the sequential input. This feature identification enables the reconstruction of unique encoding of the network when old inputs are shown to the network. New untrained classes are identified by the unsupervised STDP-WTA network that is fed by the feature map to learn the new input class. This is analogous to the hippocampal system that adapts to the new input while also recognizing previous inputs with the help of structural accumulation in the neocortex. However, the implementation utilizes pre-known labels for the training of feature identifier. This enables the system to achieve high accuracy (93% for untrained classes) but constraints the application to previously known dataset-label combinations for feature identification. The authors ensure that the feature map encodes all features uniquely by covering all possibilities within the seven untrained classes. This, however, may not be an option for continual learning agents as they may encounter input stimuli with features not previously seen. The algorithm must have the ability to identify new unseen information from older ones to be able to deal with such data.

### 3.2. Hebbian plasticity–homeostatic stability balance

Much attention is also given to Hebbian plasticity - stability balance owing to the dual-fold requirement of adapting to new data, implemented by Hebbian plasticity, while simultaneously retaining important information, implemented by homeostatic stability (Abraham and Robins, 2005). Multiple methodologies can lead to Hebbian plasticity- stability balance such as collusion of compensatory processes on multiple timescales, as shown by Zenke and Gerstner (2017). While small delays lead to higher activity and faster adaptations of weights to incoming data, large delays and refractory periods lead to lesser spiking activity and preservation of weights in the presence of local learning rules such as spike time dependent plasticity (STDP).

#### 3.2.1. Three-factor learning

Various studies suggest the role of third-factor agents, also called neuromodulators, that directly or indirectly affect plasticity, hence contributing to plasticity-stability balance (Bailey et al., 2000; Lisman et al., 2011; Gerstner et al., 2018). In the context of spiking neural networks, a global function changes the hyper-parameters of the network, such as the learning rate or decay rate of neurons, to achieve the balance when new data arrives. Recent studies have also intrigued interest in the role of hetero-synaptic plasticity that inspires global-local learning algorithms for continual and lifelong learning, as shown by Wu et al. (2022).

Wu et al. (2022) showcased a hybrid local-global meta-learning rule where modulation of weights and network parameters is done in two separate optimization levels, allowing tweaking of the network as required. Interestingly, the global learning rule updates sparse connections for learning task-specific information, whereas other connections are updated by local update rules to learn information common between the tasks. Hyper-parameters are updated after the update of synaptic weights. Considering deployment in autonomous systems, as most global optimization techniques use supervised training, the deployment gets limited to only trained tasks in a well-controlled environment, without automating the state of the neural network, whether to train or infer, as the change in the task is not automatically detected. Supervision molds the synaptic strength as required; no extra computation of the importance of weights is required while learning for the next task, but the approach supposedly demands multiple accesses to weights and neuron states due to two levels of algorithmic hierarchy, along with complex reconfiguration of artificial synapses.

Other three-factor learning algorithms, such as those proposed by Bohnstingl et al. (2019) and Stewart and Neftci (2022), emulate bi-level optimization of parameters. In the former, the outer loop algorithm responsible for the hyper-parameter update is cross-entropy based, with a very long duration required for convergence. The inner loop is implemented on neuromorphic hardware proposed by Friedmann et al. (2017). Stewart and Neftci (2022) utilized the surrogate gradient descent method, implemented on Intel's Loihi (Davies et al., 2018).

#### 3.2.2. Adaptive threshold

Other traits like the adaptive threshold of neurons have also been seen to work in coordination in maintaining the plasticity-stability balance (Muńoz-Mart́ın et al., 2020). The study by Hammouamri et al. (2022) is based on continual learning using threshold modulation. The implementation consists of two spiking networks. The network responsible for classification has its output neurons with the threshold determined by the other network, trained on a family of tasks using an evolutionary algorithm with population vectors equivalent to this network's parameters. The fitness function used for training the modulating network is the net average of the accuracy of the classification of subsequent tasks. The implementation is slow to converge owing to evolutionary training and requires labels to determine classification accuracy for several tasks.

### 3.3. Spatio-temporal sparsity

Another inherent factor in systems designed for continual learning is the spatio-temporal sparsity of activity in the network when encoding for different information. This is required to maintain independence between sub-networks encoding for old and new information. Evidently, spatial sparsity is exhibited in the brain, as shown by many studies that investigate mechanisms behind storing episodic memories (Wixted et al., 2014). For example, Wixted et al. (2018) showed that different fractions of neurons in the hippocampus show strong reactions corresponding to different words as stimuli. The remaining large fraction of neurons shows reduced firing when exposed to newer words, illustrating spatial sparsity shown by the brain in encoding for different information. Many NCL algorithms, such as those presented by Panda et al. (2018), Muńoz-Mart́ın et al. (2019), Allred and Roy (2020), Bianchi et al. (2020), and Yuan et al. (2021) utilize the winner-take-all approach at the outermost layer using excitatory-inhibitory connections for creating spatio-temporal sparsity. Allred and Roy (2020) also used non-uniform synapse modulation. Also, a fixed-size single-layer network achieved high accuracy without using supervised training. The approach utilizes competition created by lateral inhibition that creates non-uniformity in the network when exposed to a certain input, thus creating spatial sparsity. The presence of novel input is identified using self-firing dopaminergic neurons that otherwise remain inhibited. The dopaminergic neuron then stimulates all other neurons, thus making them rapidly adapt to new information. Other neurons in the network are inhibited by lateral inhibition, thus storing prior information.

### 3.4. Neurogenesis

Dynamically growing networks are inspired by neurogenesis, which occurs in the adult mammalian brain for adapting to new information (Kempermann et al., 2004).

Imam and Cleland (2020) used neurogenesis for lifelong learning in combination with other biologically inspired mechanisms implemented on Intel's Loihi (Davies et al., 2018). The study demonstrated lifelong learning while identifying odor from high-dimensional noisy olfactory signals from chemosensor arrays. A grow-when-required algorithm is implemented, introducing new nodes when a new odor is encountered. It is essential to note that the high performance of the algorithm is ensured by multiple other mechanisms incorporated in the network, such as neuromodulatory optimization of circuit properties and STDP-based local learning rule. The implementation also utilizes sparse excitatory and dense inhibitory networks that lead to the temporal encoding of the information (Buzsáki and Wang, 2012). Wang et al. (2014) presented another approach that utilizes temporal coding to encode information while adding neurons to the hidden layer as a new class arrives. Supervised STDP is proposed to train synapses between the hidden layer and the output neuron. In case all neurons delay in spiking when input is presented, a new neuron is adaptively added. Neurons with activity timing lesser than a set threshold are pruned. Hence, it inherently utilizes an unsupervised mechanism for growing adaptive structure with high classification accuracy on various datasets though not tested for incremental learning by the authors. Zhang et al. (2022), on the contrary, used the spiking activity of the neurons to determine the neurons nearest to the input and grow the network if the activity is found to be lesser than a threshold. The neurons and synapses also got removed upon inactivity for a duration greater than the threshold to regulate the network size, forgetting the previous information.

Hajizada et al. (2022) realized a neural state machine to determine the recruitment of new output neurons depending on the activity of input and output layers. The state machine is also responsible for deciding when to update the weights of the network and whether a label is to be requested. The network is implemented on Intel's Loihi.

### 3.5. Controlled forgetting

Controlled forgetting is another aspect of biological systems where the focus is to partially forget or weaken the activity corresponding to older information by identifying redundant weights. In neuroscience, Liu et al. (2022) came up with an interesting notion of “reactivation,” suggesting that forgetting does not lead to memory loss but only makes it difficult to gain the information without presenting that stimulus to invoke the memory again. Asymmetric local learning rules and excitatory and inhibitory feedback connections can be exploited to emulate controlled forgetting. Spiking neural networks developed by Panda et al. (2018) and Allred and Roy (2020) have shown to work excellently in identifying the network parts that could be forgotten to accommodate new information. Panda et al. (2018) developed a modified local learning mechanism that leaks certain weights storing insignificant information while retaining the ones storing old important information. This results in forgetting insignificant data, making the network ready to learn new tasks in a fixed network structure. This is similar to weight regularization, except the identification of insignificant information that occurs automatically. The leak time constant of the weight, which can be considered as a local hyper-parameter, is modulated based on pre and postsynaptic neuron activity instead of a global error function. The authors argue that asynchronicity helps the system to learn multiple patterns as only a few parts of the network are active at any time due to their event-driven nature, thus saving from computation overload. However, this rule also requires providing previous samples in larger quantities than later ones as the number of samples presented to the network signifies the remembering ability of the network for that class.

**Figure 1:**
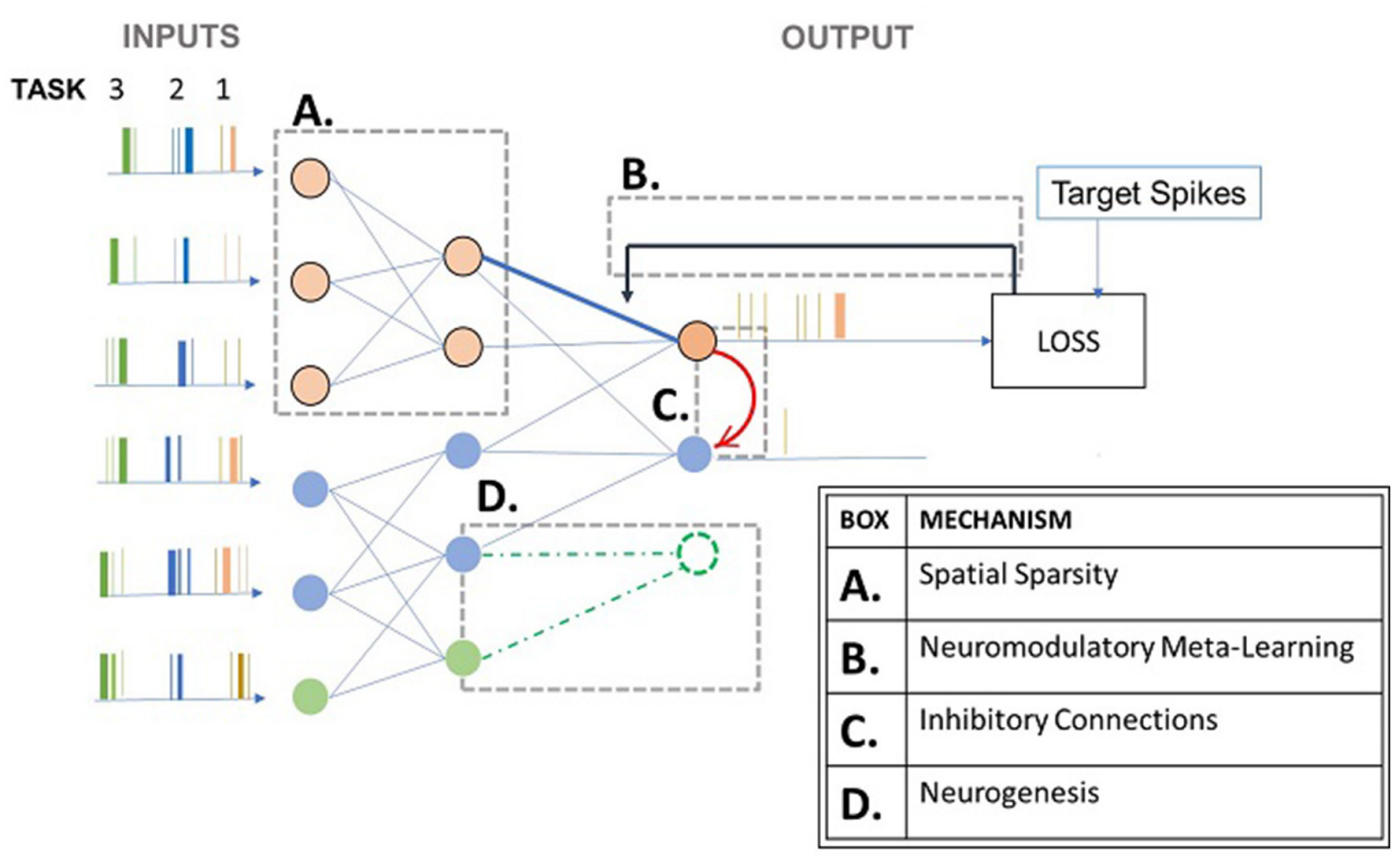
Mechanisms in spiking neural networks for continual learning. The gray boxes represent the mechanisms that are listed in the table. The figure illustrates a neural network that encounters inputs related to different tasks 1, 2, and 3 sequentially.

**Figure 2:**
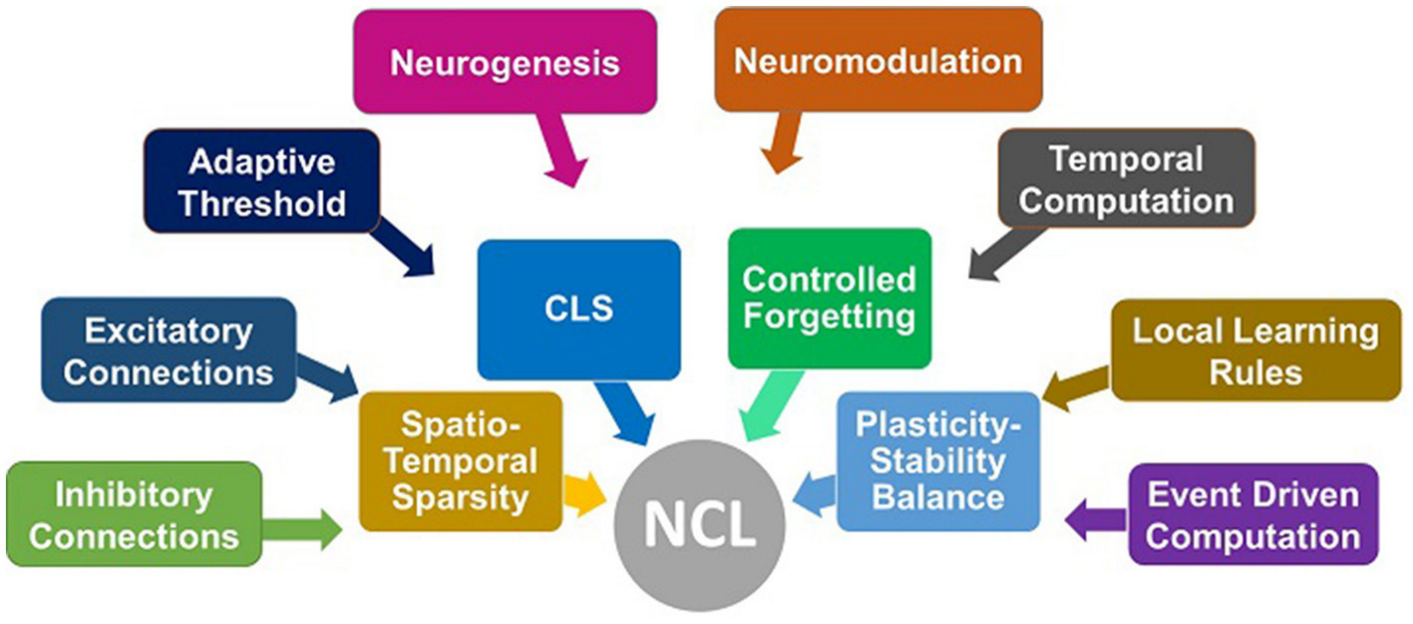
Illustration of plausible cause-effect relationship for continual learning. The causes (event driven computation, excitatory, inhibitory connections, adaptive threshold, etc.) may directly or indirectly contribute to the effects [spatio-temporal sparsity, complementary learning system (CLS), controlled forgetting, plasticity - stability balance] that ultimately lead to continual learning.

Figure 1 attempts to illustrate certain bio-inspired mechanisms seen in several studies. The spiking neural network responds to three different tasks presented to it sequentially. Box A represents the activation of a sub-network when the network encounters task 1 (Allred and Roy, 2020). Box B represents neuromodulation that is based on the loss in the case of supervised learning approaches (Wu et al., 2022). This affects the synaptic strength (Stewart and Neftci, 2022) and even hyper-parameters such as learning rate and neuron membrane potential decay as in some studies (Wu et al., 2022). Box C represents an inhibitory connection between the output neurons to emulate the winner-take-all approach so that classification can happen (Muńoz-Mart́ın et al., 2019). Box D represents the recruitment of new output neurons to train on a new task, previously not encountered by the network, as and when it gets encountered (Zhang et al., 2022). Figure 2 attempts to summarize this section by illustrating plausible algorithmic constituents in spiking neural networks that may ultimately lead to continual learning.

## 4. Hardware aspects of NCL systems

To discuss end-to-end solutions, analysis of hardware implementation holds high significance. In the continual learning scenario, critical aspects of spatio-temporal sparsity, increased network parameters requiring updates, and multi-hierarchy weight update rules pose more challenges than regular (non-continual) spiking network implementations. Some aspects worthy of consideration for hardware for NCL systems are as follows:

1. **Imbalanced data-flow**: Spatio-temporal sparsity is a critical aspect of continual learning that can cause imbalanced data-flow and difficulty in the prediction of SNN workload. The challenge of congestion of communication channels can thus arise along with difficulty in adaptive resource scheduling and mapping for on-the-fly implementation.
2. **Increased independent network state variables**: NCL algorithms rely on multiple parameters and hyper-parameter updates such as neuron threshold, decay rate variation, and axonal delays. These algorithms also require updating the membrane potential and synaptic weights as per the updated hyper-parameters. These updates may lead to an increase in memory access per timestep, consuming energy and slowing processing. These updates may also require highly reconfigurable in-memory computing paradigms with a high-performing interface.
3. **Support of different neuronal models and learning rules**: While various platforms are able to support different learning rules, incorporating the programmability of a neuron model directly affects the design of finite state machines implementing the sequential neuron parameter updates. While the incorporation of modules such as microcode functionality that enable such programmability is progressing (Orchard et al., 2021), there is still a long way to go to be able to achieve a general architecture for highly programmable neuronal models and update rules.
4. **Multi-timestep neuronal parameter update**: A constraint of multi-timestep neuronal membrane potential update for emulating LIF dynamic causes interference with general dataflow scheduling for synaptic weight update in simultaneously occurring local learning rules.

In the further sub-sections, we discuss hardware implementations of NCL systems in the current literature. We discuss how these implementations are able to mitigate or work around the above issues. Two types of neuromorphic hardware implementations are utilized by current NCL systems, namely, digital neuromorphic architectures and custom nano-device-based architectures. Both follow similar parallel distributed systems but differ in the amount of near-memory and in-memory computation supported. While the former supports high programmability, the latter tailors the system for higher energy efficiency. Both are compared on relevant parameters and are shown to support different algorithms with differing strengths. Certain other approaches are discussed that open up directions for further development of hardware specifically for supporting continual learning approaches.

### 4.1. Digital hardware platforms used by NCL systems in the literature

In digital neuromorphic platforms, re-programmability is one key aspect, along with parallel “cores” and local memory access to obtain the benefit of energy efficiency. However, bottlenecks arrive in two aspects. First, the neuro-core shared by various neurons generally shares the same parameters, such as time constants for membrane updates and threshold potentials. Second, the number of fan-ins and fan-outs of one neuron is restricted due to the localization of computation. Table 1 enumerates key aspects of digital neuromorphic architectures as reported in the literature.

**Table 1:** Comparison of various neuromorphic platforms.

**Features**	**LOIHI**	**TIANJIC**	**HICANN**	**SPINNAKER**
Technology	14nm Finfet	28nm HLP CMOS	65nm CMOS	130nm CMOS
Number of parallel neurons accessible per step	1K	Extendable (Pei et al., 2019)	64	-
Event routing mechanism	Mesh routing	Reconfigurable LUT based, adjacent multicast	Wired, with switches at intersection	NOC
Communication protocol	AER	Non AER	AER	AER
Near memory computing supported	Yes	Yes	Yes	No
NCL algorithm implemented	Surrogate gradient based three factor	Hybrid global–local meta learning rule	Inner loop of L2l (reinforcement learning)	STDP, SRDP, custom
Weight storing device	SRAM	Locally stored in SRAM	SRAM	SDRAM, DTCM
Neuron models supported	LIF, ADExp	LIF, ANN	AdExp, LIF (Schemmel et al., 2010)	Point neuron, LIF, Izhekevich
Weight precision	9-bit	8-bit weights	Analog	22 bits (Stromatias et al., 2015)

The table attempts to provide insight on the architectural make-up of implementations.

An algorithm such as three-factor learning, as proposed by Stewart et al. (2020), has been implemented on Intel's Loihi (Davies et al., 2018). Neuro-cores provide the processing required for neuronal and synaptic updates. The chip utilizes multiple cores with parallel accessibility to 1 million neurons in one timestep per core. Activity scheduling is done using a dedicated scheduler for multiple spiking activities at the same timestep. The hierarchical routing mechanism helps in dealing with the multi-chip mapping of the implemented neural network. Multiple compartments are used in the implementation to emulate the three-factor learning rule using the learning engine provided by the cores. x86 cores ensure the programmability of learning rules.

Hajizada et al. (2022) present another NCL algorithm implemented on Loihi. It uses the local learning engine of the processor for online continual learning as the learning is localized to a single layer for object recognition.

Certain hardware implementations like Tianjic (Deng et al., 2020) unify spiking and non-spiking network emulation, making it easier to implement global functions for the three-factor-learning scenario. Wu et al. (2022) have implemented the system on Tianjic and utilized its many-core architecture.

Another implementation emulating the inner loop of three-factor learning is HICANN (Friedmann et al., 2017) used by Bohnstingl et al. (2019). The system utilizes a microprocessor for executing learning rules and synaptic updates. This ensures reconfigurability. The implementation is mixed-signal as the neurons are emulated using analog circuits. The algorithm, however, is tested for transfer learning in reinforcement learning tasks and not for catastrophic forgetting. Certain aspects of the architecture are elucidated in Table 1 for comparison with other architectures.

Mikaitis et al. (2018) emulated the three-factor STDP learning rule and dopaminergic neurons on SpiNNaker. The architecture of SpiNNaker incorporates 18 ARM cores per chip, connected via network on chip. This architecture thus allows flexibility in the implementation of neurons, synapses, connections, and learning rules while trading off power and resource utilization (Stromatias et al., 2013). While the algorithm emulated is three-factor STDP, the system, however, has not been tested on the continual learning scenario.

Narayanan et al. (2020) designed a system that does computation only when activity occurs by utilizing the same resource. The activity is timestamped in the current layer being executed. The same hardware is utilized for next-layer computations. While it attempts to save computational resources, it has a high databus requirement for retrieving the corresponding weights for membrane potential update. It is also difficult to implement connections within the same layer.

To reduce memory footprint, certain approaches utilize approximations such as dyadic function (Karia et al., 2022). Certain others simplify neuronal models by replacing inhibiting neurons with lateral inhibitory connections to avoid complex neuronal updates (Putra and Shafique, 2021). Putra and Shafique (2021) proposed an algorithm to find spiking neuron models that optimize energy consumption and memory footprint. The approach however requires knowing the input samples to be processed to make the memory-energy consumption estimate.

Another important factor is the parameter precision trade-off. A higher resolution is required as the majority of weights take values close to extremes. High bit width impacts memory print. With low bit widths, accuracy of the algorithm is hugely affected. To overcome this, Karia et al. (2022) have used dual fixed point formats that incorporate both using a mode bit set high for representing high precision and low for representing a high dynamic range. Li et al. (2022) utilized a mixed precision scheme, with floating-point representation for rapidly changing synaptic weights and binary values for holding onto information as they do not go under much effect upon activation.

### 4.2. Nano-Device-based emerging architectures

Nano-Device-based emerging architectures focus on the in-memory computation of network functions such as leaky integrate and fire emulation as well as complex synaptic weight updates. The approach promises higher energy efficiency as more computation is incorporated into the memory module, removing data movement requirements for processing (Kim et al., 2015; Luo and Yu, 2021). These implementations also have parallel implemented “cores” (Jiang et al., 2019), but most of the presented studies in the literature for NCL systems are highly custom-tailored for the algorithm being implemented. For example, Muńoz-Mart́ın et al. (2019) implemented STDP-WTA network with phase change memory devices as synaptic elements. The WTA functionality is implemented using a transistor between pre and post-neuron. This makes it difficult to implement any change in the algorithm once the structure gets hardwired.

Furthermore, another device-specific implementation is done by Yuan et al. (2021). In this study, authors have shown dynamically expanding networks for incremental learning. The advantage of the approach comes with seamless gate tunability offered by a memtransistor which simplifies addressability in crossbar arrays and efficiently enables tunable learning rules and bio-realistic functions (Yan et al., 2022). While programming energy is critical in these implementations, certain devices ensure low-energy operation due to inherent programming conditions. In the study presented by Chekol et al. (2021), the state is reversed as the programming voltage reaches 0.2 V, with current leakage of less than 10fA, where a memtransistor requires 30 V of programming voltage (Yuan et al., 2021).

Apart from this, non-volatile devices present inherent challenges of non-uniformity in cycle-to-cycle and device-to-device variation. As an ingenious workaround, many studies exploit the inefficacy of the devices for emulating algorithmic components (Suri et al., 2013b; Kumar et al., 2016). For example, Shaban et al. (2021) incorporated non-ideality in the algorithm implementation where the approach is based on adaptive thresholding. The threshold adaptively changes as the neuron is exposed to a new task. The setup uses a custom circuit that incorporates an OxRAM crossbar array for emulating the neuron circuit. Muñoz-Martin et al. (2021) exploited the conductance drift of the phase change memory (PCM) device for active forgetting. The authors devise a neuron with internal homeostatic and plastic regulation, inherently achieving stability-plasticity balance. They incorporate these neurons in a hybrid supervised-unsupervised network that resembles a complimentary learning system. The network is designed for navigation tasks where the agent learns from rewards and penalties during environment exploration. Lim et al. (2021) showed that resistance drift observed in phase change devices improves convergence as the resistance drifts in the high-resistance state. Sparsification, pruning, and quantization are proposed at the algorithmic end to bridge the gap between algorithmic requirements and hardware performance. Other studies, such as the one presented by Suri et al. (2013a), have shown drift resilience using in-memory architectural workarounds such as differential synaptic cells to cancel out the effect of drift.

**Figure 3:**
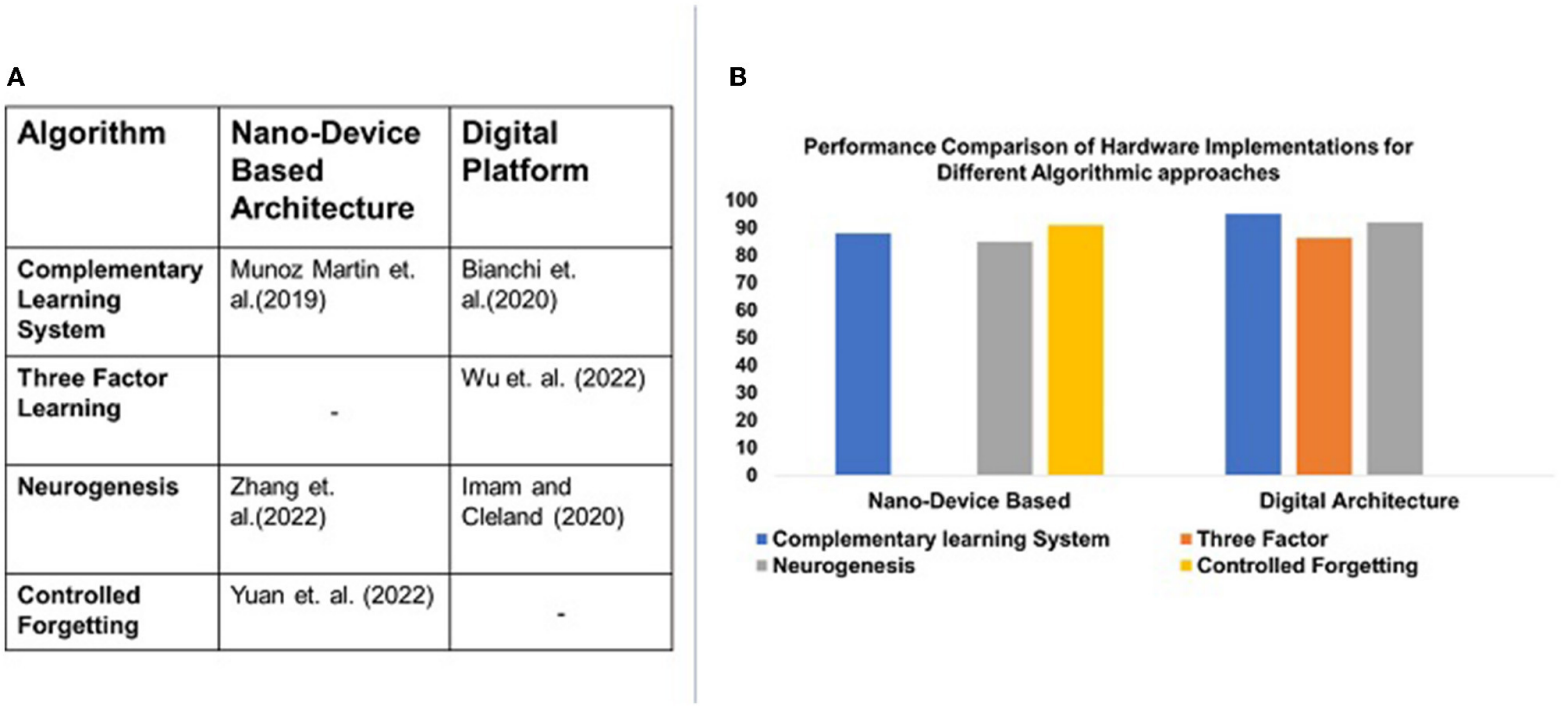
Illustration of NCL algorithms supported by current architectures. **(A)** Illustrates the gap in the current literature on algorithm-hardware mapping. Algorithms such as three factor learning have not yet been implemented using nano-device architectures. Controlled forgetting algorithms are not yet implemented with digital platforms. Their accuracy is compared in **(B)**.

While nano-device-based architectures provide energy efficiency, to the best of our knowledge, no NCL system emulating three-factor learning has been presented in the literature. Furthermore, while digital platforms provide a vast range of algorithmic emulation, controlled forgetting is not evident in the literature. Figure 3A illustrates the gap in algorithm-hardware mapping, as discussed above. Figure 3B illustrates the performance of these systems emulating respective algorithms, as reported in the literature.

## 5. Performance evaluation metric for NCL systems

D́ıaz-Rodŕıguez et al. (2018) proposed a metric, termed as “*CL*_*score*_” (Continual Learning Score) or “$CL_{score}^{ANN}$” for the benefit of the below discussion and analyzed artificial neural network approaches for continual learning using this metric. To calculate the $CL_{score}^{ANN}$, D́ıaz-Rodŕıguez et al. (2018) calculated the weighted sum of several criteria that evaluate the performance of ANN approaches. Some of the criteria can be adopted as-is for assessing neuromorphic systems, namely “accuracy,” “backward transfer,” and “model size efficiency.” But due to underlying differences between ANN and SNN-based approaches, specifically in the domain of learning rules and hardware implementation, we propose the below-mentioned modifications for calculating “$CL_{score}^{SNN}$”for enabling assessment of NCL systems:

1. **Computational Efficiency (CE)**: D́ıaz-Rodŕıguez et al. (2018) proposed this criterion which calculates computational efficiency using a number of multiply and accumulate operations in forward and backward pass of the network to learn a task using back-propagation. Most neuromorphic approaches utilize local learning paradigms instead of back-propagation (Allred and Roy, 2020; Wu et al., 2022). Such learning paradigms are represented by a number of synaptic operations and membrane potential updates in multiple studies (Davies et al., 2018; Deng et al., 2020). Hence, we minorly modified the criteria as shown below:
  (1) $$CE=min(1,\frac{\sum_{i=1}^{N}\frac{OP_{INF}(T_{i})}{OP_{TRAIN}(T_{i})}}{N}),$$
  where *OP*_*INF*_(*T*_*i*_) and *OP*_*TRAIN*_(*T*_*i*_) are the number of synaptic operations and membrane potential updates per timestep for inference and training for the *i*^*th*^ task, respectively.
2. **Sample Storage Size efficiency (SSS)**: While many ANN-based approaches utilize a memory replay-based mechanism, neuromorphic approaches have been shown to avert this mechanism (Muńoz-Mart́ın et al., 2019; Allred and Roy, 2020; Yuan et al., 2021). However, multiple approaches depend on pre-trained sub-network in NCL systems to achieve high accuracy (Muńoz-Mart́ın et al., 2019; Bianchi et al., 2020; Hammouamri et al., 2022; Stewart and Neftci, 2022). The pre-trained sub-network stores prior information in the form of learned weights while it is trained offline on pre-known stimulus. Hence, we propose to consider memory utilization by pre-trained sub-network as given below:
  (2) $$\begin{array}{l} SSS=1-min\left(1,\frac{M_{pretrained}}{M_{total}}\right), \end{array}$$
  where *M*_*pretrained*_ is the number of neurons and synapses of the pre-trained sub-network and *M*_*total*_ is the total number of neurons and synapses in the complete network.
3. **Energy Efficiency (EE)**: We propose energy efficiency as an additional criterion for the calculation of $CL_{score}^{SNN}$. It attempts to highlight the energy efficiency of multiple neuromorphic hardware architectures emulating NCL algorithms over a conventional computing platform such as CPU, emulating SNN. The calculation of the criteria is given below:
  (3) $$\begin{array}{l} EE=1-min\left(1,\frac{E_{NCL}}{E_{CPU}}\right), \end{array}$$
  where *E*_*NCL*_ is the energy consumption per timestep by the neuromorphic hardware while emulating the NCL algorithm and *E*_*CPU*_ is energy consumed per timestep by the CPU, emulating a two-layer, fully connected SNN, as reported by Parker et al. (2022), who also benchmarked the energy efficiency of neuromorphic platform against the reported CPU platform.

The metric, $CL_{score}^{SNN}$, is calculated using the weighted sum of the above criteria:


(4) $$\begin{array}{l} CL_{score}^{SNN}=\sum w_{i}\times C_{i}, \end{array}$$

where *C*_*i*_ ∈ (accuracy, backward knowledge transfer, model size efficiency, sample storage size efficiency, computational efficiency, and energy efficiency) and $w_{i}=\frac{1}{\#C}$

Table 2 elucidates $CL_{score}^{SNN}$ of the studies discussed in Sections 3 and 4. The studies are also assessed based on pre-existing metrics such as energy per SOP and average test accuracy. Analysis of NCL systems based on average test accuracy alone showcases studies such as the one proposed by Bianchi et al. (2020) to be efficient on singular tasks. This metric however cannot provide a measure of catastrophic forgetting in the network and energy efficiency (D́ıaz-Rodŕıguez et al., 2018). Analysis of studies based on Energy per SOP highlights studies such as Zhang et al. (2022). However, the metric of Energy per SOP alone may not quantify catastrophic forgetting and computational efficiency of the implementations in continual learning tasks. The analysis of studies using the modified metric, $CL_{score}^{SNN}$, on the contrary, assesses the performance of NCL systems in multiple aspects simultaneously and highlights studies such as Imam and Cleland (2020), Wu et al. (2022), and Zhang et al. (2022). These studies perform well in terms of multiple criteria, such as backward transfer of knowledge and energy consumption during re-training of the network. Adaptation of bio-inspired mechanisms in an intricate manner is seen in studies such as Imam and Cleland (2020) that may help the network achieve computational efficiency along with high performance in continual learning tasks. For example, Imam and Cleland (2020) utilized temporal encoding using excitatory-inhibitory networks and dynamically expanded the network when required by the task.

**Table 2:** Benchmarking of NCL systems on the basis of average test accuracy, energy per SOP, and $CL_{score}^{SNN}$.

**Type**	**Work**	**Task**	**Hardware**	**Average Test Accuracy**	**Energy Per SOP (pJ)**	** $CL_{score}^{SNN}$ **
CLS	Muńoz-Mart́ın et al. (2019)	Image Classification (MNIST)	Nano-device Based (PCM)	93%	1*	0.60
	Bianchi et al. (2020)	Image Classification (MNIST)	FPGA	**97%**	-	0.49
TFL	Wu et al. (2022)	Image Classification (MNIST)	Tianjic	86.3%	1.5	**0.75**
DEN	Imam and Cleland (2020)	Odour Classification (Vergara et al., 2013)	Loihi	92%	105.3	**0.73**
	Zhang et al. (2022)	Image Classification (MNIST)	Nano-Device Based (Perovskite Nickelate)	85%	**0.002**	**0.67**
CF	Yuan et al. (2021)	Image Classification (MNIST)	Nano-Device Based (MoS_2)	92%	1.06 × 10^6^	0.65

CLS, complementary learning system; TFL, three-factor learning; DEN, dynamically expanding networks; CF, controlled forgetting. *Bianchi et al. (2019).

**Figure 4:**
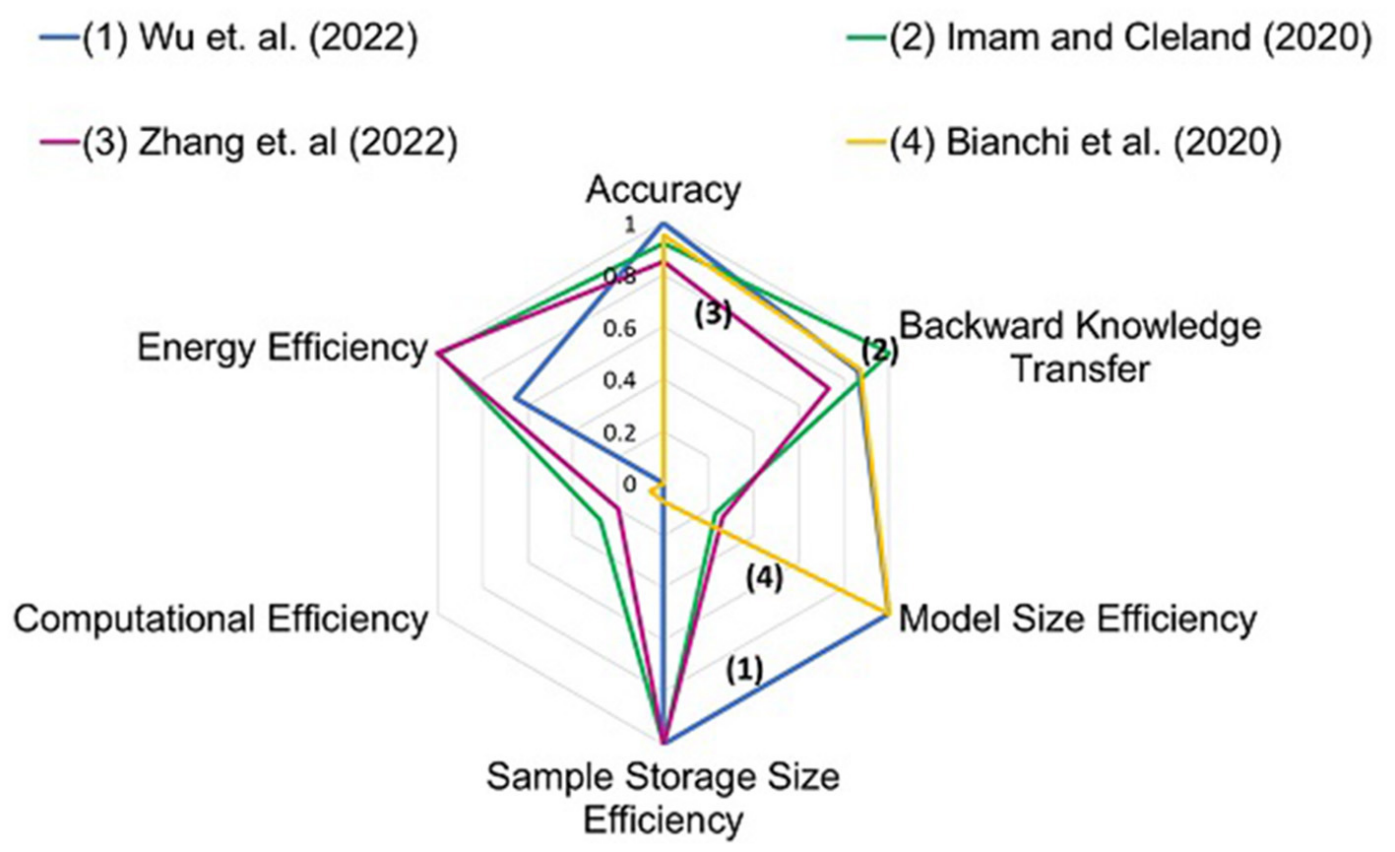
Radar plot for different studies. Four studies with different hardware platforms and NCL algorithms are plotted. The studies show differing strengths in different criteria. Numbers are mentioned against each study in the graph for identification.

Wu et al. (2022) synergistically incorporated both local and global learning. Implementation on hybrid ANN-SNN emulator (Deng et al., 2020) ensures low-energy consumption along with efficient emulation. Zhang et al. (2022) emulated a grow-when-required scheme, implemented using Perovskite Nickelate, with very low programming energy. Figure 4 elucidates the performance of different studies in different criteria of the metric. More area signifies better overall performance. The calculation of energy for each task is given in Table 3. This estimation is done for the calculation of energy efficiency criteria in Section 5.

**Table 3:** Estimating energy consumption of the system.

**Work**	**Energy consumption (mJ)**	**Remarks**
Muńoz-Mart́ın et al. (2019)	1.7	Training power (Bianchi et al., 2020) × Timestep (s) (Muńoz-Mart́ın et al., 2019)
Bianchi et al. (2020)	18.5	Training power (Bianchi et al., 2020) × Timestep (s) (Muńoz-Mart́ın et al., 2019)
Wu et al. (2022)	3.46	Reported energy per inference (latency = 1 timestep)
Imam and Cleland (2020)	0.00215	Reported energy per inference / timesteps per inference
Zhang et al. (2022)	0.000004	Estimated programming energy per device × estimated devices programmed per timestep
Yuan et al. (2021)	714.6	Estimated programming energy per device × estimated devices programmed per timestep

The estimation is based on different component values reported in each study, with the method of calculations provided in the remark section of the table.

## 6. Discussion

This section discusses the roadmap for further development of NCL systems. The research and co-optimization are discussed at all abstraction levels. Finally, the conclusion summarizes the key takeaways from the study.

### 6.1. Road ahead for NCL systems

There are certain cues in biological systems not yet adapted for continual learning applications. Such approaches include dendritic computation (Stöckel and Eliasmith, 2021) and context-dependent gating (Tsuda et al., 2020). Studies such as those proposed by Liang and Indiveri (2019) and Yang et al. (2021) are based on hierarchical context-dependent gating but have not been tested against continual learning tasks.

From the algorithmic viewpoint, complementary learning system-based algorithms currently lack in adaptively identifying a structure in an ensemble of items fed to the neuromorphic system to emulate neo-cortical function. In three-factor learning, algorithms do not identify sub-networks responsible for encoding important information for previous tasks. Hence, while Hebbian plasticity-stability balance is maintained, while simultaneous spatio-temporal sparsity is not, possibly causing the utilization of larger network sizes.

In most algorithms, winner-take-all learning is implemented using supposedly delicately balanced excitatory and inhibitory connections. Fault tolerance in critical stages can make the excitatory and inhibitory circuits enter a non-desirable state, possibly stepping away from continual learning. Designing should be done while also covering the undesirable states that should converge to idle states. Very few implementations utilize time-based coding schemes that can save on resources. The possibility of initiating faster dynamics for the network adapting to newer information while tuning the remaining network to a slow setting to avoid change in synaptic weights in local learning scenarios has not been investigated well.

Algorithms used for predicting network activity for effective hardware resource mapping and scheduling have to be developed further at a low computational cost. They also have to be dynamic in their predictions with changing network activity in the continual learning scenario. Many resource mapping and scheduling techniques are built for spiking convolutional networks. There is a need to design techniques for balancing workloads for continual learning algorithms. Most resource mapping and workload predicting algorithms today may not be taking continual learning workloads into consideration and are not continually adaptive (Song et al., 2022). Chen et al. (2022) have attempted to mitigate workload imbalance by dividing channels into subgroups of equal workload, where workload has been calculated proportionally to weights. These algorithms have not yet been tested on continual learning workloads. Another approach can be distributing the workload based on differing time constants throughout the network that emulates fast and slow adaptation to data. Another method to map the resources can be to assign specific processing elements to modules that identify as and when new information arrives and map the remaining resources to the active sub-network accordingly, which is not yet implemented in the literature.

Population-based neural mapping techniques also need to be tailored such that they can incorporate variable resetting thresholds and other important parameters for continual learning. Xiang et al. (2017) proposed an approach in this direction, which is yet to be tested on continual learning workloads. Another drawback appears to arise from the extra processing space that the algorithm takes to constantly reduce the spurious weight updates for saving energy.

At the device level, while in-memory computation is lucrative, it can lead to large and power-consuming peripheral circuitry, repeated across the cores for multi-core architecture, along with complicated and algorithm-specific circuit design. Functionally, in the context of continual learning, studies presented by Muńoz-Mart́ın et al. (2020) and Shaban et al. (2021) have paved the way for an efficient implementation of adaptive threshold incorporation within devices, proven to be beneficial for continual learning (Hammouamri et al., 2022), but implementation for continual learning tasks is yet to be seen at the device level for these algorithms. Similarly, studies presented by Muliukov et al. (2022) have integrated self-organizing map functionality on ReRAM and FeFets but have not yet tested the implementation on continual learning tasks. Moreover, ensuring reconfigurable logic mapping by the NCL hardware for implementing various algorithms is important. The development of hybrid architectures is arguably necessary to incorporate system-level nuances of in-memory computing modules for end-to-end deployment.

**Figure 5:**
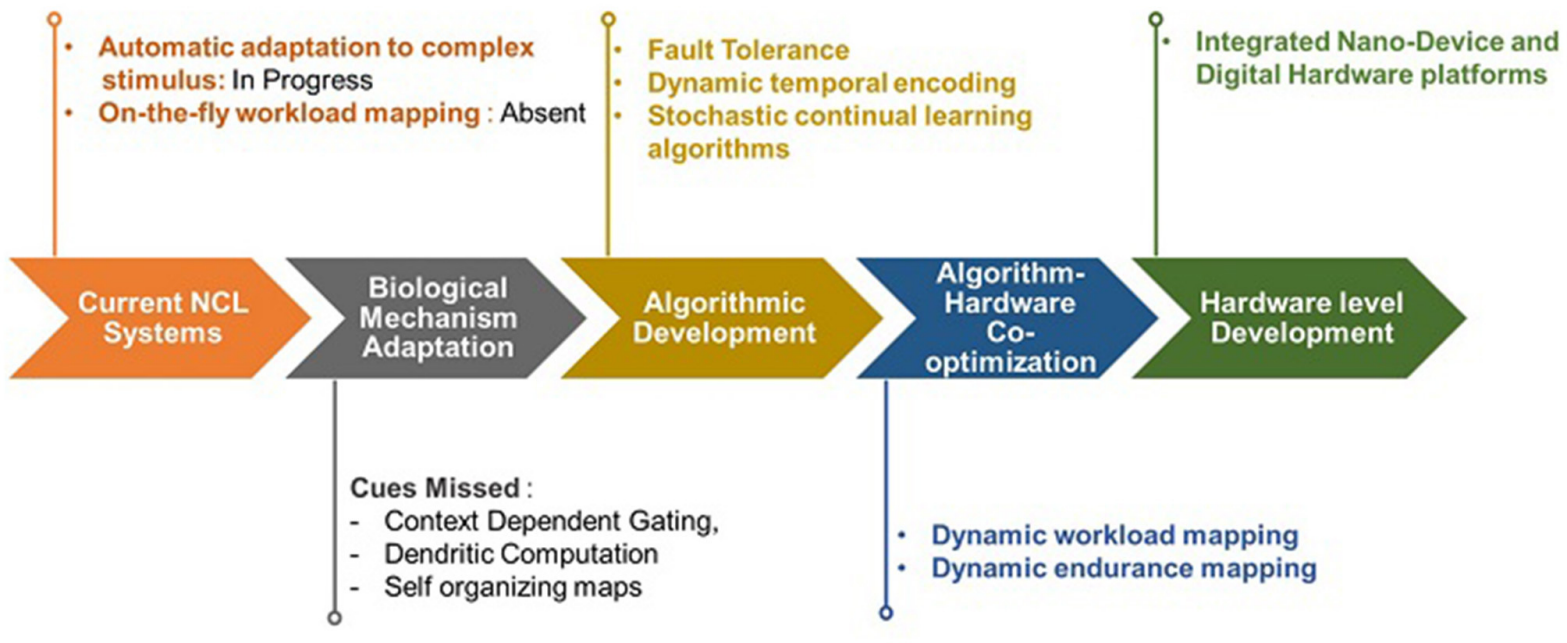
Roadmap for NCL Systems. The figure illustrates plausible directions that can be taken at multiple abstraction levels to develop NCL systems for deployment in real-world applications.

Figure 5 illustrates a summary of a plausible roadmap for further development of NCL systems.

### 6.2. Conclusion

In this review, we discussed mechanisms for neuromorphic continual learning based on algorithms present in current literature. We discussed important hardware considerations and workaround to support NCL algorithms. We analyzed different studies based on the modified metric for continual learning systems. We identified the neuromorphic approaches utilized by works functioning well on this metric. We also identified research gaps throughout the analysis and proposed a roadmap for further development of NCL systems.

## Author contributions

All authors listed have made a substantial, direct, and intellectual contribution to the work and approved it for publication.
